# Myocarditis: Which Role for Genetics?

**DOI:** 10.1007/s11886-021-01492-5

**Published:** 2021-05-07

**Authors:** Chiara Baggio, Giulia Gagno, Aldostefano Porcari, Alessia Paldino, Jessica Artico, Matteo Castrichini, Matteo Dal Ferro, Rossana Bussani, Marco Merlo

**Affiliations:** 1 Cardiothoracovascular Department, Azienda Sanitaria Universitaria Giuliano Isontina (ASUGI) and University of Trieste, Via Valdoni 7, 34129 Trieste, Italy; 2 Barts Heart Centre, St Bartholomew’s Hospital, London, UK; 3 Cardiothoracic Department, Institute of Pathological Anatomy and Histology, Azienda Sanitaria Universitaria Giuliano Isontina (ASUGI), University of Trieste, Via P. Valdoni 7, 34100 Trieste, Italy

**Keywords:** Myocarditis, Genetics, Inflammatory cardiomyopathies, Pathogenic mutations, Genetic testing, Post-inflammatory dilated cardiomyopathy

## Abstract

**Purpose of Review:**

Myocarditis is a polymorphic disease, both in its presentation and clinical course. Recent data suggests that the genetic background, interacting with environmental factors, could be diriment both in the susceptibility and evolution of myocarditis in different clinical presentations. The aim of this paper is to expose the current available evidences and the evolving concepts on this topic, in order to provide insight for improving the clinical management of those patients. In this regard, the main goal is an optimal characterization of each patient’s risk, with the purpose of individualizing the treatment and the follow-up.

**Recent Findings:**

The latest research highlights the possible prognostic role of some pathogenic mutations that could create a vulnerable myocardium prone to myocardial inflammation and also to the development of a long-lasting cardiomyopathy.

**Summary:**

The identification of these genetic defects and of myocarditis patients requiring genetic testing is emerging as a challenge for the future. In fact, identifying a possible genetic background responsible for a particularly high-risk profile could be of extreme importance in improving management of myocarditis. This and many other aspects in the genetics of myocarditis remain uncovered, and further studies are expected based to refine our daily clinical practice.

## Introduction

Myocarditis is a polymorphic disease characterized by a great variability both in its clinical presentation and evolution [1••].

Many efforts have been made to find useful tools for identifying patients with a high-risk and a poor prognosis. While there have been many advances in this field, there are still numerous unsolved issues. With the growing evidence of the role of genetics in other cardiovascular diseases, such as cardiomyopathies [2–4], one recently raised and still unanswered question is if there could be a role for genetics also in myocarditis.

This review aimed at gathering the evidences available on this topic and examining the perspectives for the future, to yield insights for a better clinical management of those patients.

## Genetic Involvement in the Pathophysiology of Myocarditis

Myocardial inflammation can result from different infectious and non-infectious causes, including viral or bacterial agents, immunological disorders, and drug toxicity. Notably, the underlying cause of the myocardial inflammation can often remain unknown [5].

Viruses account for approximately 90% of myocarditis. Three stages of viral infection have been postulated: phase 1, viral entry into myocytes and activation of innate immunity; phase 2, viral replication and activation of acquired immune responses; and phase 3, evolution toward resolution with recovery or development of dilated cardiomyopathy (DCM) [6].

Although genome sequences of more than 27 viruses have been detected in hearts with myocarditis, only the pathogenic mechanism of enteroviruses has been well studied in animal models. This pathogenic mechanism implies the internalization through a transmembrane Coxsackievirus and adenovirus receptor and the induction of a rapid cytolysis with subsequent cardiac inflammation [6–10]. Mechanisms of viral entry, replication, cellular injury, and death from other non-enteroviruses remain poorly understood and are still a matter of investigation [10].

In clinical practice, the question regarding why some patients develop myocarditis with different clinical severity and presentation, and other subjects exposed to the same interfering environmental factors (mostly viruses) do not, has been frequently raised. Data suggests that there may be a genetic predisposition toward the development of the disease [11•].

It is established that a key role in the pathophysiology of myocarditis is played by a maladaptive response of the immune system to specific environmental triggers [6, 12]. Since specific genetic loci have been discovered to determine different immune responses against infections, it has been postulated that genetic heterogeneity could help in understanding the diverse individual susceptibility to myocarditis. Thus helping in understanding why, despite the same environmental exposure, only specific individuals may develop myocarditis with different clinical manifestation. Available evidences regarding immune reactions and genetically defined host factors are conflicting as emerged from a recent study by Belkaya et al., where the authors failed to demonstrate a relationship between *TLR3* and *STAT1* deficiency and an increased susceptibility to viral myocarditis [13, 14]. Furthermore, single gene variants related to myocarditis are rare, and the available information is based on single case reports and small clinical series. More frequently, a genetic condition leading to immunodeficiency could act as a predisposition for the occurrence of myocarditis [15]. Therefore, more research is needed to identify constitutional gene variants which can influence the development of myocardial inflammation when exposed to environmental triggers.

Another debated issue is whether the genetic heterogeneity could also explain the different attitude toward a rapid viral clearance and resolution/chronic evolution of the myocardial inflammation. The attitude toward a rapid viral clearance or toward viral persistence may be determined also by the type of virus and the individual immune response. Furthermore, it is possible that a given insult (i.e., virus) produces an injury responsible of an inflammatory activation that in turn triggers the pathogenic mutant proteins further inciting injury and inflammation in a vicious circle. Therefore, inflammation may persist through a mechanism that is now independent of virus persistence. This may differentiate those subjects with and without genetic mutations. Finally, the viral clearance is only one of the components of the complex interplay between genetic background and environment that remains in general widely unexplored and represents the target of future research.

## Genetic Involvement in the Clinical Presentation and Evolution of Myocarditis

The heterogeneity of clinical presentation of myocarditis ranges from subclinical or benign forms that are generally characterized by chest pain as the main presenting symptom (i.e., low-risk forms) to major clinical syndromes, such as severe heart failure or life-threatening ventricular arrhythmias (i.e., high-risk forms) [16]. Whether genetics can play a role in determining the development of a definite clinical presentation over another is still unclear, and few evidences are known.

### High-Risk Myocarditis

It seems that the development of a dysregulated inflammatory response (characterized by elevated levels of cytokines like *IL-1beta*, *IL-17*, and *TNF* and imbalance between metalloproteinase and their inhibitors) which is linked to higher risk clinical presentation could be genetically determined as it happens in patients with the mutation of the major histocompatibility complex genes, in particular *HLA-DR4* [17]. If there are other high-risk genotypes underlying, a severe presentation is still a matter of investigation.

Patients presenting with high-risk myocarditis (i.e., heart failure or life-threatening arrhythmias associated to left ventricular (LV) dysfunction) are usually characterized by a poorer prognosis. However, there is an important variability in individual natural history, with some patients fully recovering and others progressing to the development of DCM [1••].

Recent studies have focused on defining if genetic variants and potentially pathogenic mutations could play a role in determining the evolution of myocarditis favoring either the progression toward DCM or, diversely, its complete resolution [18•]. Notably, potentially harmful polymorphisms of the genes responsible for genetically determined cardiomyopathies appear to be correlated to the development of post-myocarditis DCM [18•, 19]. In this view, a complex interplay between predisposing factors and the inflammatory insult could be at the basis of different clinical course of the disease. This complex interaction involves not only genes responsible for the viral infection itself but also genes encoding for structural proteins, through an interaction between viral proteases and cytoskeleton proteins, thus predisposing to the development of a long-lasting LV dysfunction after the acute inflammatory event [18•, 19].

It has been postulated that genetic defects in structural proteins create a vulnerable myocardium prone to myocardial seeding by a pathogen, thus favoring the persistence and progression of myocarditis [11•]. In this sense, the mutation of *Dystrophin* predisposes its cleavage from viral proteases, resulting in increased susceptibility to sarcomere rupture, more rapid virus propagation, higher viral titers, and greater cardiomyopathy [20]. Moreover, the new highlighted role of altered miRNA profile determined by the virus in lymphocytic myocarditis may concur to the unfavorable evolution of the inflammatory insult. Conversely, mutations affecting genes codifying for non-structural proteins (like *SCN5A* and *BAG3*) seem to portend to a more favorable progression of the disease [21].

Recently, an innovative report highlighted that in patients with biopsy-proven myocarditis, especially if presenting with HF and LV dysfunction, almost 30% of cases are carrying pathogenic or likely pathogenic variants for cardiomyopathy causing genes [19]. In particular, *Titin* was the most prevalent mutation in patients presenting with myocarditis and LV systolic dysfunction and was associated with lower rate of recovery over time [19].

In this scenario, it is still unclear whether the myocarditis is a transitional stage to overt DCM or if it represents a bystander manifestation of a genetic mutation/polymorphism. For instance, in specific settings, myocardial viral infection might exacerbate the underlying cardiomyopathy and specifically be the trigger for the progression of a Duchenne DCM [22]. Furthermore, there is increasing evidence that myocarditis may be the first manifestation of other forms of cardiomyopathy, such as arrhythmogenic cardiomyopathy (AC). In recent years, few cases have been reported of genetically determined cardiomyopathies whose first clinical presentation was that of an acute, often uncomplicated, myocarditis. For these cases, it is still debated whether the viral/immune inflammatory process acts as the initiator of the myocardial injury, being therefore the fibro-fatty infiltration the result of the healing process, or if myocarditis remains a distinct disease that mimics AC. Also, AC due to *desmoplakin (DSP)* mutation was found to be associated with intermittent myocardial inflammatory episodes clinically similar to those of myocarditis. From this angle, myocarditis seems to be part of the clinical presentation in the natural history of the disease leading to an arrhythmogenic phenotype in genetically predispose patients [23]. That is why perhaps cardiotropic viruses are more frequently identified in patients with AC than in control subjects. Interestingly, in patients with *DSP* mutation and recurrent myocarditis, intense physical activity has been described as another potential trigger, thus reinforcing the need to consider genomic-environment interaction [24].

DCM and AC are two different forms of structural cardiomyopathy, where the first one is mostly characterized by a dilated left (and possibly right) ventricle with a reduced systolic function, while in the second, the main feature is the fibro-fatty replacement favoring the development of malignant arrhythmias. Despite being often described as two different entities, DCM and AC present several overlapping aspects. In the context of myocarditis, it is not clear whether one patient may evolve to DCM rather than AC. However, it seems that this different evolution could be driven by the genetic background, with the mutation in structural proteins leading most commonly to DCM and desmosomial pathogenic variants determining the development of AC. Moreover, there can be the case of a patient with an already established, asymptomatic cardiomyopathy with a superimposed myocarditis. Data on this condition are not available, so it is not yet known whether this new inflammatory injury is responsible of a worsening of the underlying cardiomyopathy in a sort of step-wise evolution of the disease. It is possible that acute inflammatory episodes trigger the expression of the pathogenic mutant protein but this has to be proved, and further studies are needed to clarify this aspect.

### Low/Intermediate Risk Myocarditis

Patients presenting with chest pain and normal ventricular function, with no wall motion abnormalities and with a stable arrhythmic profile, seem to have an excellent prognosis [25]. On the other hand, for patients with chest pain associated with wall motion abnormalities, mild ventricular dysfunction, or persistent ECG abnormalities, the prognosis is still uncertain [1••]. There have been only few studies focusing on possible prognostic markers in this group of patients, and, for instance, it has been observed that the anterior-septal CMR LGE localization [26] and the presence of an early LV remodeling at mid-term follow-up could correlate with a worse prognosis, particularly in terms of life-threatening arrhythmias experience [27]. However, the role of genetics in patients at low/intermediate risk has not been investigated yet and remains widely obscure. Therefore, future large and multicenter studies should focus on clarifying whether specific genetic backgrounds could correlate with different clinical presentations of myocarditis and, most importantly, if it could be a predictive factor of an unfavorable prognosis. In fact, the finding of particular “high-risk genetic backgrounds” would be of great importance in the development of an optimal patient-tailored therapy and follow-up. This would pave the way to new studies focusing on the possible interaction between the genetic background and environment modifiers (such as viruses) in order to find new strategies to improve the prognosis of myocarditis in individuals with a specific genetic setting.

**Fig. 1:**
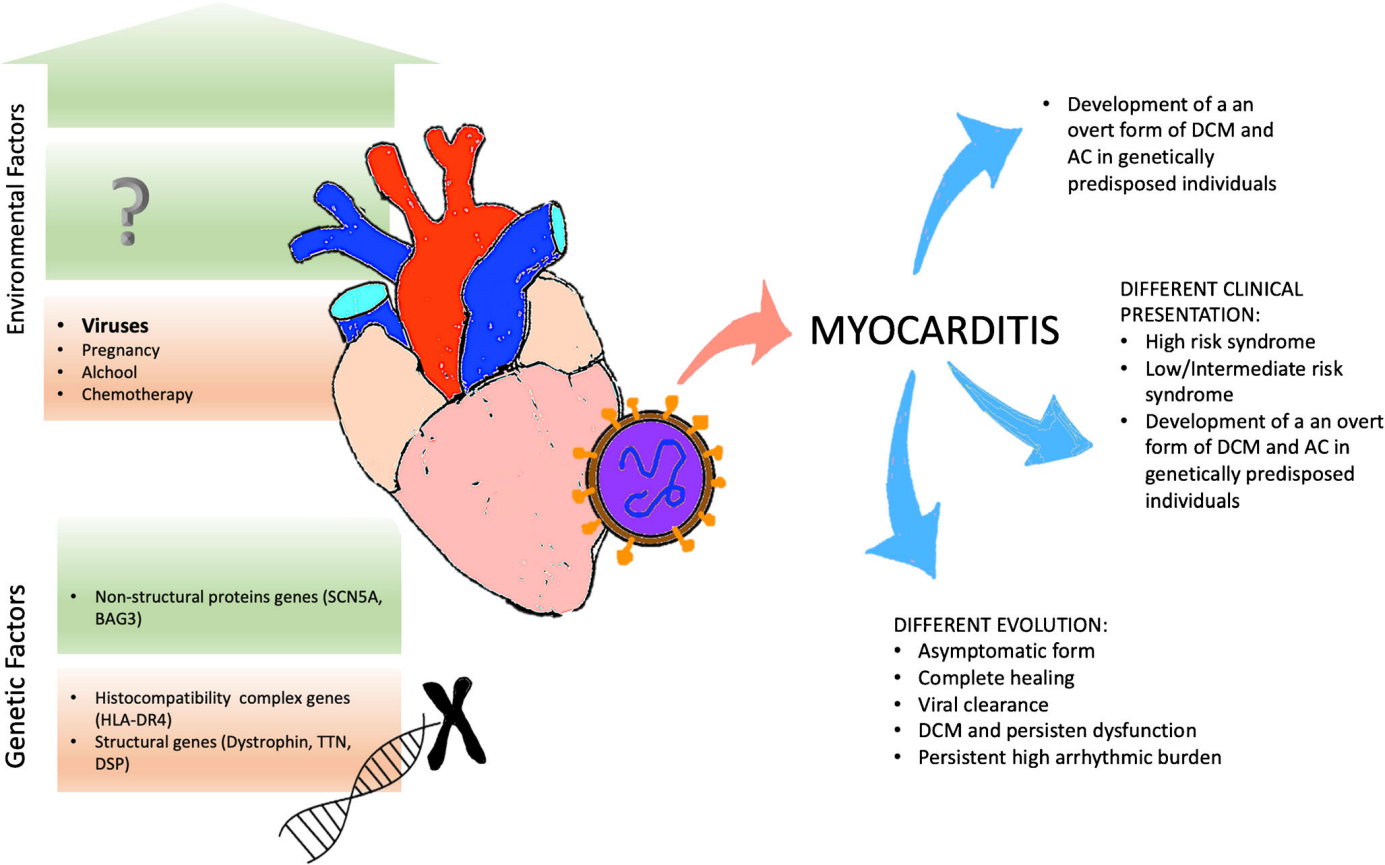
Schematic representation of the genetic involvement in the pathophysiology and evolution of myocarditis. Protective environmental and genetic factors are listed in *green boxes*, while adverse genetic and environmental factors are listed in *red boxes*. The development of a clinical evident form of myocarditis and the eventual evolution towards inflammatory dilated cardiomyopathy rather than the complete virus clearance with no or only mild clinical manifestation is determined by the complex interplay between the genetic background and superimposed environmental factors. AC, arrhythmogenic cardiomyopathy; DCM, dilated cardiomyopathy

Figure 1 summarizes the main aforementioned concepts.

## TTN Truncating Variants: an Example of the Interaction Between Genetics and Environment

The background of cardiomyopathies is quite broad, and, in recent times, the complex interaction between genetic mutations and environmental factors helped unveil a wide range of conditions to better understand the pathophysiology of cardiomyopathies. In particular, *Titin (TTN)* is one of the most common mutation of DCM, and it is estimated that *TTNtv* may account for up to one-third of familial DCM cases [2]. In recent years, the idea that this gene possibly does not directly cause the cardiomyopathy but instead it acts as a modifier needing a second environmental hit to arise has emerged. On the other hand, increasing evidences point out that some of known secondary forms of cardiomyopathy, such as the alcohol-induced cardiomyopathy, the peripartum, the chemotherapy-induced, and in specific setting also the myocarditis, might have a genetic mutation on the background, which will predispose the development of the clinical phenotype. In particular, the peripartum cardiomyopathy shares a similar genetic background with the DCM, showing in approximately one-third of cases a genetic mutation, mostly *TTNtv*, leading to the phenotype [4]. Similarly, in patients with excess alcohol intake, having a *TTNtv* mutation predisposes to a more severe phenotype of the disease characterized by larger diameters and lower ejection fraction compared to those without the predisposing mutation [3]. In both conditions, the variants detected in the population were found with a frequency similar to that seen in DCM, suggesting a common pathophysiological background that can justify, or partially explain, the development of the disease. In addition, unrecognized rare variants in cardiomyopathy-associated genes, particularly *TTNtv*, have been shown to increase the risk for chemotherapy-induced cardiomyopathy in children and adults and adverse cardiac events in adults [28]. Curiously, it has recently been reported [19] that also in a cohort of adult patients with biopsy-proven lymphocytic myocarditis, the genetic yield was similar, especially for *TTNtv* prevalence, to a geographically comparable cohort of sporadic DCM [29]. This suggests that the inflammatory insult on the heart might uncover an increased genetic susceptibility to develop overt LV dysfunction or arrhythmogenic phenotypes.

## Genetic Analysis in Patients with Myocarditis: Which Role in Clinical Practice?

While it seems reasonable to suspect a genetic basis both in the development and clinical course of myocarditis, there is still a lack of recommendation when performing genetic testing in this field. Similar to what it has just been reported for DCM [30–32], genetic basis of myocarditis may be suspected in the presence of specific “red flags” either in personal or in family history. Indeed, family history of cardiomyopathy, sudden cardiac death, and pacemaker implantation in early age may suggest the transmission of pathogenic genetic variants. Also, the recurrence of acute myocarditis has been recognized as a clear risk factor for underling genetic mutation [15, 24]. Moreover, genetic predisposition to myocarditis is also supported by the presence of clinical traits at the physical examination (e.g., neurosensory disorders, skeletal muscle involvement, woolly hair, and keratoderma), at the laboratory analysis (e.g., creatine kinase elevation), at the ECG evaluation (e.g., persistent left bundle branch block, AV block, posterolateral pseudonecrosis, low voltage, epsilon wave), and at cardiac RMN (e.g., diffuse LGE).

Finally, it has to be clearly stated that a negative family history does not rule out a genetic predisposition to myocarditis, due to the presence of possible de novo mutations and of incomplete penetrance. In this context, persistent LV systolic dysfunction during follow-up may be an indicator of underlying genetic variant in cardiomyopathy-related genes. In fact, as recently reported by Artico et al. [19], a genetic analysis of patients with persistent LV dysfunction or arrhythmias after an episode of acute myocarditis revealed that a consistent proportion of them was a carrier of a pathogenic variant in sarcomeric or desmosomal genes.

To date there is no indication for performing routinely a genetic test in patients with a diagnosis of myocarditis. Genetic testing might be considered in the presence of clinical “red flags” that should be carefully and systematically evaluated (see Table 1), and in persistent LV dysfunction or malignant arrhythmias during follow-up. In all cases, when a pathogenic variant in non-immunity genes coding for structural proteins whose defects trigger heritable cardiomyopathies is identified, clinical and genetical family screening is mandatory [31].

**Table 1:** Clinical red flags suggesting genetic testing in patients with myocarditis

“Red flags”	Suggested causes
Family history of cardiomyopathy, sudden cardiac death, pacemaker implantation	
Clinical history and physical examination	
Mental retardation	Dystrophinopathies; mitochondrial diseases
Neurosensory disorders	Mitochondrial diseases
Skeletal muscle involvement	Dystrophinopathies; desminopathies; laminopathies
Woolly hair and keratoderma	Carvajal syndrome
Pregnancy	Peripartum DCM
Recurrence of acute myocarditis	AC
Laboratory analysis	
Increased creatine kinase	Dystrophinopathies; desminopathies; myofibrillar myopathy; laminopathies
ECG	
Atrio-ventricular blocks	Laminopathies; desminopathies
Low voltages	Filaminopathies
Posterolateral pseudonecrosis	Dystrophinopathies
T negative waves in V1-3 <14yo, or V1-4 >14yo	AC
Epsilon wave	AC
Echocardiography	
Posterolateral akinesia	Dystrophinopathies
Cardiac hypertrophy	Infiltrative heart diseases
RV dyskinesia/akinesia/aneurysm	AC
Cardiac magnetic resonance	
Adipose infiltration	AC
Diffuse LGE	AC
RV dyskinesia/akinesia/aneurysm	AC

*AC* arrhythmogenic cardiomyopathy, *DCM* dilated cardiomyopathy, *LGE* late gadolinium enhancement, *RV* right ventricular

## Conclusions

In conclusion, there are growing evidences on the role of genetics both in the susceptibility and evolution of myocarditis, especially in patients presenting with severe LV dysfunction and evolution to DCM. However, these evidences need further future confirmation through multicenter focused studies based on larger populations, before being validated in daily clinical practice. Furthermore, many aspects in the genetics of myocarditis remain uncovered. In particular, data are lacking in patients with intermediate-low-risk syndromes, with chronic myocarditis and in patients with high arrhythmic burden. Therefore, future efforts should focus on filling these gaps, in order to better characterize all patients with myocarditis, paving the way to a more and more individualized and risk-tailored approach.

## References

[1] •• Sinagra G, Anzini M, Pereira NL, et al. Myocarditis in clinical practice. Mayo Clin Proc. 2016. 10.1016/j.mayocp.2016.05.013**This paper resumes the current knowledge about myocarditis and daily clinical practice**. 10.1016/j.mayocp.2016.05.013 27489051

[2] Herman DS, Lam L, Taylor MRG, Wang L, Teekakirikul P, Christodoulou D, Conner L, DePalma SR, McDonough B, Sparks E, Teodorescu DL, Cirino AL, Banner NR, Pennell DJ, Graw S, Merlo M, di Lenarda A, Sinagra G, Bos JM, Ackerman MJ, Mitchell RN, Murry CE, Lakdawala NK, Ho CY, Barton PJR, Cook SA, Mestroni L, Seidman JG, Seidman CE (2012). Truncations of titin causing dilated cardiomyopathy. N Engl J Med.

[3] Ware JS, Amor-Salamanca A, Tayal U, Govind R, Serrano I, Salazar-Mendiguchía J, García-Pinilla JM, Pascual-Figal DA, Nuñez J, Guzzo-Merello G, Gonzalez-Vioque E, Bardaji A, Manito N, López-Garrido MA, Padron-Barthe L, Edwards E, Whiffin N, Walsh R, Buchan RJ, Midwinter W, Wilk A, Prasad S, Pantazis A, Baski J, O’Regan DP, Alonso-Pulpon L, Cook SA, Lara-Pezzi E, Barton PJ, Garcia-Pavia P (2018). Genetic etiology for alcohol-induced cardiac toxicity. J Am Coll Cardiol.

[4] Ware JS, Li J, Mazaika E, Yasso CM, DeSouza T, Cappola TP, Tsai EJ, Hilfiker-Kleiner D, Kamiya CA, Mazzarotto F, Cook SA, Halder I, Prasad SK, Pisarcik J, Hanley-Yanez K, Alharethi R, Damp J, Hsich E, Elkayam U, Sheppard R, Kealey A, Alexis J, Ramani G, Safirstein J, Boehmer J, Pauly DF, Wittstein IS, Thohan V, Zucker MJ, Liu P, Gorcsan J, McNamara D, Seidman CE, Seidman JG, Arany Z, IMAC-2 and IPAC Investigators (2016). Shared genetic predisposition in peripartum and dilated cardiomyopathies. N Engl J Med.

[5] Trachtenberg BH, Hare JM (2017). Inflammatory cardiomyopathic syndromes. Circ Res.

[6] Pollack A, Kontorovich AR, Fuster V, Dec GW (2015). Viral myocarditis-diagnosis, treatment options, and current controversies. Nat Rev Cardiol.

[7] Mahfoud F, Grtner B, Kindermann M (2011). Virus serology in patients with suspected myocarditis: utility or futility?. Eur Heart J.

[8] Sinagra G, Porcari A, Gentile P, Artico J, Fabris E, Bussani R, et al. Viral presence-guided immunomodulation in lymphocytic myocarditis: an update. Eur J Heart Fail. 2020. 10.1002/ejhf.1969. 10.1002/ejhf.1969 PMC7405140 32683758

[9] Shi Y, Chen C, Lisewski U, Wrackmeyer U, Radke M, Westermann D, Sauter M, Tschöpe C, Poller W, Klingel K, Gotthardt M (2009). Cardiac deletion of the Coxsackievirus-adenovirus receptor abolishes coxsackievirus b3 infection and prevents myocarditis in vivo. J Am Coll Cardiol.

[10] Pankuweit S, Klingel K (2013). Viral myocarditis: from experimental models to molecular diagnosis in patients. Heart Fail Rev.

[11] Campuzano O, Fernández-Falgueras A, Sarquella-Brugada G, Sanchez O, Cesar S, Mademont I, Allegue C, Mates J, Pérez-Serra A, Coll M, Alcalde M, Iglesias A, Tiron C, Gallego MÁ, Ferrer-Costa C, Hospital A, Escribano C, Dasí C, Borondo JC, Castellà J, Arbelo E, Medallo J, Brugada J, Brugada R (2015). A genetically vulnerable myocardium may predispose to myocarditis. J Am Coll Cardiol.

[12] Heymans S, Eriksson U, Lehtonen J, Cooper LT (2016). The quest for new approaches in myocarditis and inflammatory cardiomyopathy. J Am Coll Cardiol.

[13] Chapman SJ, Hill AVS (2012). Human genetic susceptibility to infectious disease. Nat Rev Genet.

[14] Belkaya S, Kontorovich AR, Byun M, Mulero-Navarro S, Bajolle F, Cobat A, Josowitz R, Itan Y, Quint R, Lorenzo L, Boucherit S, Stoven C, di Filippo S, Abel L, Zhang SY, Bonnet D, Gelb BD, Casanova JL (2017). Autosomal recessive cardiomyopathy presenting as acute myocarditis. J Am Coll Cardiol.

[15] Arbustini E, Narula N, Giuliani L, Di Toro A. Genetic basis of myocarditis: myth or reality? In: Myocarditis. 2020:45-89. 10.1007/978-3-030-35276-9_4.

[16] Anzini M, Merlo M, Sabbadini G, Barbati G, Finocchiaro G, Pinamonti B, Salvi A, Perkan A, di Lenarda A, Bussani R, Bartunek J, Sinagra G (2013). Long-term evolution and prognostic stratification of biopsy-proven active myocarditis. Circulation..

[17] Li HS, Ligons DL, Rose NR (2008). Genetic complexity of autoimmune myocarditis. Autoimmun Rev.

[18] Cannata A, Artico J, Gentile P, Merlo M, Sinagra G (2019). Myocarditis evolving in cardiomyopathy: when genetics and offending causes work together. Eur Heart J Suppl.

[19] Artico J, Merlo M, Delcaro G, Cannatà A, Gentile P, de Angelis G, Paldino A, Bussani R, Ferro MD, Sinagra G (2020). Lymphocytic myocarditis: a genetically predisposed disease?. J Am Coll Cardiol.

[20] Xiong D, Lee GH, Badorff C, Dorner A, Lee S, Wolf P, Knowlton KU (2002). Dystrophin deficiency markedly increases enterovirus-induced cardiomyopathy: a genetic predisposition to viral heart disease. Nat Med.

[21] Knowlton KU (2017). Myocarditis: an intersection between genetic and acquired causes of human cardiomyopathy. J Am Coll Cardiol.

[22] Mavrogeni S (2015). Cardiac involvement in Duchenne and Becker muscular dystrophy. World J Cardiol.

[23] Alley R, Grizzard JD, Rao K, Markley R, Trankle CR. Inflammatory episodes of desmoplakin cardiomyopathy masquerading as myocarditis: unique features on cardiac magnetic resonance imaging. JACC Cardiovasc Imaging. 2020. 10.1016/j.jcmg.2020.07.028. 10.1016/j.jcmg.2020.07.028 32950456

[24] Poller W, Haas J, Klingel K, Kühnisch J, Gast M, Kaya Z, Escher F, Kayvanpour E, Degener F, Opgen-Rhein B, Berger F, Mochmann HC, Skurk C, Heidecker B, Schultheiss HP, Monserrat L, Meder B, Landmesser U, Klaassen S (2020). Familial recurrent myocarditis triggered by exercise in patients with a truncating variant of the desmoplakin gene. J Am Heart Assoc.

[25] Ammirati E, Cipriani M, Moro C, Raineri C, Pini D, Sormani P, Mantovani R, Varrenti M, Pedrotti P, Conca C, Mafrici A, Grosu A, Briguglia D, Guglielmetto S, Perego GB, Colombo S, Caico SI, Giannattasio C, Maestroni A, Carubelli V, Metra M, Lombardi C, Campodonico J, Agostoni P, Peretto G, Scelsi L, Turco A, di Tano G, Campana C, Belloni A, Morandi F, Mortara A, Cirò A, Senni M, Gavazzi A, Frigerio M, Oliva F, Camici PG, On behalf of the Registro Lombardo delle Miocarditi (2018). Clinical presentation and outcome in a contemporary cohort of patients with acute myocarditis multicenter Lombardy registry. Circulation..

[26] Aquaro GD, Perfetti M, Camastra G, Monti L, Dellegrottaglie S, Moro C, Pepe A, Todiere G, Lanzillo C, Scatteia A, di Roma M, Pontone G, Perazzolo Marra M, Barison A, di Bella G, Cardiac Magnetic Resonance Working Group of the Italian Society of Cardiology (2017). Cardiac MR with late gadolinium enhancement in acute myocarditis with preserved systolic function: ITAMY study. J Am Coll Cardiol.

[27] Filippetti L, Mandry D, Venner C, Juillière Y, Sadoul N, Girerd N, Lamiral Z, Selton-Suty C, Marie PY, Huttin O (2018). Long-term outcome of patients with low/intermediate risk myocarditis is related to the presence of left ventricular remodeling in addition to the MRI pattern of delayed gadolinium enhancement. JACC Cardiovasc Imaging.

[28] Garcia-Pavia P, Kim Y, Restrepo-Cordoba MA, Lunde IG, Wakimoto H, Smith AM, Toepfer CN, Getz K, Gorham J, Patel P, Ito K, Willcox JA, Arany Z, Li J, Owens AT, Govind R, Nuñez B, Mazaika E, Bayes-Genis A, Walsh R, Finkelman B, Lupon J, Whiffin N, Serrano I, Midwinter W, Wilk A, Bardaji A, Ingold N, Buchan R, Tayal U, Pascual-Figal DA, de Marvao A, Ahmad M, Garcia-Pinilla JM, Pantazis A, Dominguez F, John Baksi A, O’Regan DP, Rosen SD, Prasad SK, Lara-Pezzi E, Provencio M, Lyon AR, Alonso-Pulpon L, Cook SA, DePalma SR, Barton PJR, Aplenc R, Seidman JG, Ky B, Ware JS, Seidman CE (2019). Genetic variants associated with cancer therapy-induced cardiomyopathy. Circulation..

[29] Gigli M, Merlo M, Graw SL, Barbati G, Rowland TJ, Slavov DB, Stolfo D, Haywood ME, Dal Ferro M, Altinier A, Ramani F, Brun F, Cocciolo A, Puggia I, Morea G, McKenna WJ, la Rosa FG, Taylor MRG, Sinagra G, Mestroni L (2019). Genetic risk of arrhythmic phenotypes in patients with dilated cardiomyopathy. J Am Coll Cardiol.

[30] Paldino A, De Angelis G, Merlo M (2018). Genetics of dilated cardiomyopathy: clinical implications. Curr Cardiol Rep.

[31] Hershberger RE, Lindenfeld J, Mestroni L, Seidman CE, Taylor MRG, Towbin JA (2009). Genetic evaluation of cardiomyopathy-a Heart Failure Society of America practice guideline. J Card Fail.

[32] Merlo M, Cannatà A, Gobbo M, Stolfo D, Elliott PM, Sinagra G (2018). Evolving concepts in dilated cardiomyopathy. Eur J Heart Fail.

